# Anti-Epcam Aptamer (Syl3c)-Functionalized Liposome for Targeted Delivery Of Doxorubicin: In Vitro And In Vivo Antitumor Studies in Mice Bearing C26 Colon Carcinoma

**DOI:** 10.1186/s11671-020-03334-9

**Published:** 2020-05-07

**Authors:** Mohammad Mashreghi, Parvin Zamani, Seyedeh Alia Moosavian, Mahmoud Reza Jaafari

**Affiliations:** 1 Nanotechnology Research Center, Pharmaceutical Technology Institute, Mashhad University of Medical Sciences, Mashhad, Iran; 2 Department of Pharmaceutical Nanotechnology, School of Pharmacy, Mashhad University of Medical Sciences, Mashhad, Iran; 3 Department of Medical Biotechnology, Faculty of Medicine, Mashhad University of Medical Sciences, Mashhad, Iran; 4 Biotechnology Research Center, Pharmaceutical Technology Institute, Mashhad University of Medical Sciences, Mashhad, Iran

**Keywords:** EpCAM, Aptamer, Liposomes, Doxorubicin, Colon cancer

## Abstract

In this study, we have surface-functionalized PEGylated-nanoliposomal doxorubicin (DOX) with anti-EpCAM (epithelial cell adhesion molecule) aptamer via post-insertion of anti-EpCAM aptamer-conjugated DSPE-mPEG_2000_ into Caelyx® (ED-lip). The size, charge, release profile, and cytotoxicity and cellular uptake of formulation were determined. The characterization of the ED-lip demonstrated the slightly increase in size and PDI along with the decrease in zeta potential which indicated that post-insertion efficiently done. The results of flow cytometry and fluorescent microscopy have shown that ED-lip enhanced the rate of cell uptake on C26 cell line compared to Caelyx®. The ED-lip also had more cytotoxic effects than Caelyx® which indicated the efficacy of anti-EpCAM aptamer as targeting ligand. The pharmacokinetic and tissue biodistribution of formulations in mice bearing C26 tumors demonstrated that ED-lip did not affect the distribution profile of DOX compared to Caelyx® in animal model. In addition, ED-lip effectively improved the tumor accumulation of DOX and promoted survival of animals compared to Caelyx®. These results suggest that the functionalization of Caelyx® with anti-EpCAM aptamer is promising in cancer treatment and merits further investigation.

## Introduction

Nano drug delivery systems (NDDSs) with the size of 100–200 nm are passively accumulated in the tumor microenvironment via enhanced permeability and retention (EPR) effect. This occurs through the loose endothelial lining and weak lymphatic drainage. However, recent data indicated that only less than 1% of administrated drug could reach to the tumor site [1]. Lack of the ability to penetrate in the dense extra cellular matrix (ECM) of tumor, return of the released drug to circulation and heterogeneity of tumors are the reasons responsible for this failure [2]. Different strategies have been used to improve tumor accumulation of NDDSs using endogenous and exogenous stimuli [3]. These NDDSs could response to the exogenous stimuli such as light and have the capacity to use in tumor imaging [4]. There are many different inorganic nanomaterials which have the ability to use as anticancer agents [5, 6]. However in case of inorganic nanomaterials attention must be paid to their toxicities and environmental safeties [7–11].

Active targeted delivery is important approach that helps NDDSs to deliver therapeutic agents more efficient to the tumors and minimizing the exposure to non-target tissues [12, 13]. An ideal targeting agent for targeted delivery is a molecule that have affinity to the cell surface proteins or receptors upregulated by particular cells or tissue components [14].

The epithelial cell adhesion molecule (EpCAM) is a transmembrane glycoprotein that considered as a candidate ligand for active targeting. Recent findings indicated that the EpCAM has normal low expression healthy epithelial cells, while in cancer cells its expression become in higher levels (up to 1000-fold) [15–17]. During the cancer development the expression pattern of EpCAM change from basal and basolateral membrane in normal epithelial to the apical surface in tumor epithelial cells [18]. This differential expression makes EpCAM as a very interesting ligand for drug delivery which could improve the therapeutic index of drug [19].

EpCAM is demonstrated as cancer stem cell (CSC) or tumor initiating cell (TIC) marker, which its expression in cancer is related to the poor prognosis [20]. CSC or TIC are cells which have self-renewal, the ability to produce more cells of the same types, that have key roles in tumor development and metastasis [21]. The overexpression of EpCAM has been reported in CSC of various solid tumors [22]. Recently, aptamers have attracted much attention in widespread range of investigation and emerge as a potentially powerful molecules that can be used in NDDSs as the targeting ligand [23, 24]. Aptamers are DNA or RNA based oligonucleotides sequences that possess secondary and tertiary structures that have affinity to their targets such as cell surface receptors [23, 24]. Aptamers also have several advantages over the for example, they are non-immunogenic and have low molecular weight (8–25 kDa) with chemical and thermal stability. In addition, their synthesis and chemical modifications is low-cost and scalable [25]. The selective targeting of the NDDSs through anti-EpCAM-specific aptamer could be considered as an effective targeting option to deliver chemotherapeutic agents into the tumor microenvironment [19, 26]. In this regard, different studies shown that anti-EpCAM aptamer functionalized nanocarriers could effectively improve the delivery of anticancer drugs to tumor cells [15, 27, 28].

**Fig. 1:**
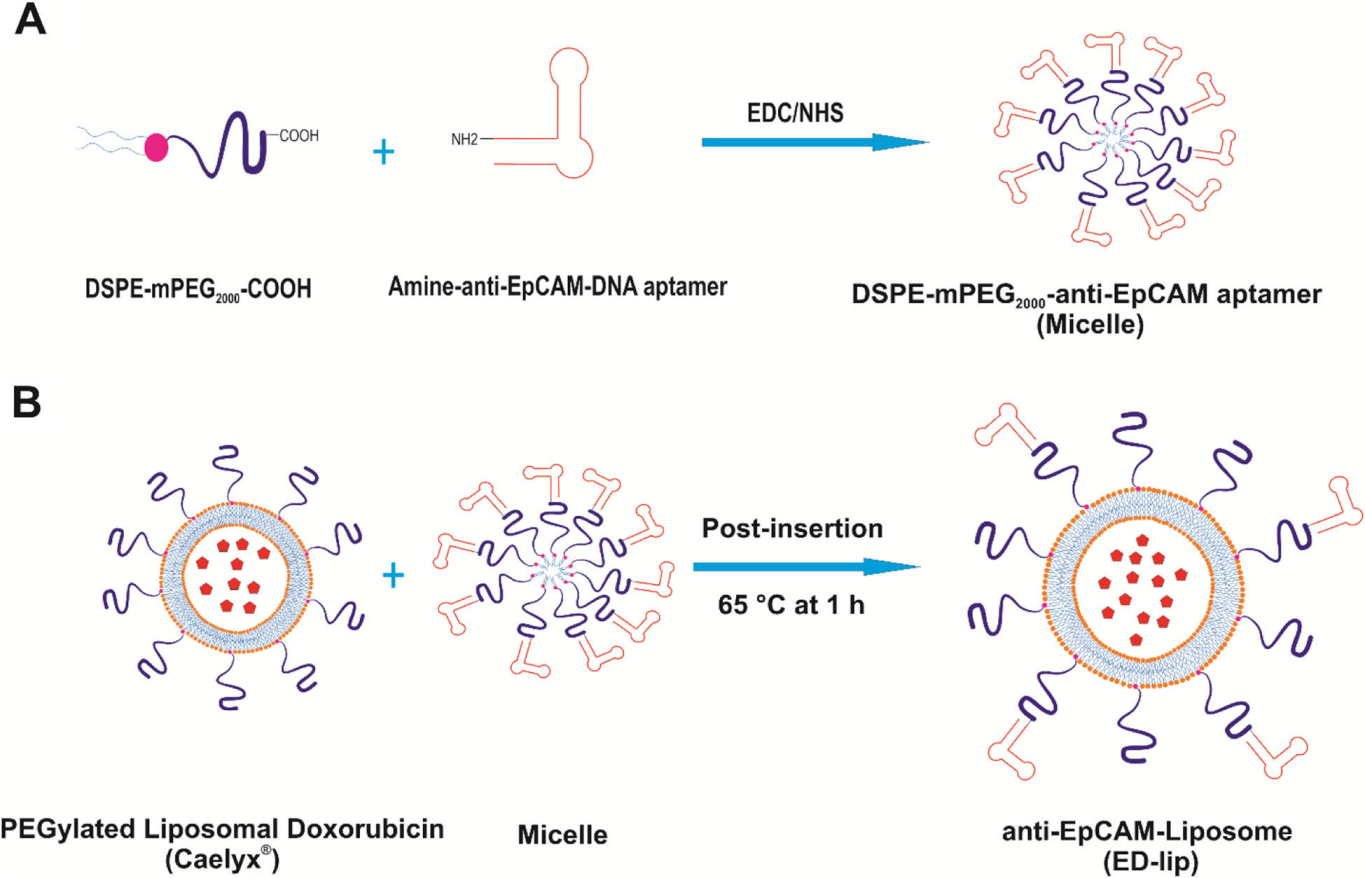
Preparation of anti-EpCAM-functionalized Caelyx® (ED-lip). **a** Linking of anti-EpCAM aptamer to DSPE-mPEG_2000_ through covalent binding of the primary amines (-NH2) of anti-EpCAM aptamer to the carboxyl group (-COOH) of DSPE-mPEG_2000_. **b** Post-insertion method for preparation of anti-EpCAM-functionalized Caelyx® (ED-lip)

The goal of this study is to develop an anti-EpCAM DNA aptamer (SYL3C)-PEGylated-nanoliposomes loaded with doxorubicin (DOX) (ED-lip) as a model of NDDS. Such functionalization was performed by EDC/NHS coupling chemistry between amine group of aptamer and carboxyl group of DSPE-mPEG_2000_, which is post-inserted into the liposome as shown in Fig. 1. The ED-lip characterized for size, zeta potential, and percentage of doxorubicin encapsulation, release profile and cytotoxicity. Then, we evaluated whether these ED-lip could improve cell uptake in vitro and deliver DOX to the tumor with via targeting in mice bearing C26 colon carcinoma tumors.

## Result and Discussion

Caelyx®, PEGylated liposomal doxorubicin is one of the most widely used chemotherapeutic agent and is the first FDA approved nanoparticle that has been indicated for treatment of ovarian cancer, AIDS-related Kaposi's sarcoma and multiple myeloma. Caelyx® passively penetrated to the tumor site via EPR effect [29]. Although, Caelyx® significantly has improved pharmacokinetics and half-life of DOX; however, the main limitations of Caelyx® are insufficient cellular uptake and low release rate of drug in tumor site [29]. Here, we used SYL3C aptamer as a targeting ligand to functionalize liposomal doxorubicin (ED-lip) to target EpCAM molecule in the surface of cancer cells, which enables the delivery of DOX to specific target site through the process of active targeting.

### Conjugation of DSPE-mPEG_2000_ to Aptamer

In the present study, we used EDC/NHS coupling chemistry for conjugation of amine functionalized anti-EpCAM aptamers to active carboxylic group of DSPE-mPEG_2000_-COOH. The advantage of this coupling reaction using EDC/NHS coupling chemistry and formation of the amide bond is its stability and reducing nonspecific interactions among aptamers [30]. Aptamers could be modified with primary amine or thiol group and covalently conjugated to activate carboxyl or pyrrole group of maleimide, respectively [31]. Aptamers modified with thiol group were conjugated to the maleimide functional group of DSPE-PEG_2000_. Then, DSPE-PEG_2000_-aptamer post-inserted into a the liposome structure to decorated the outer surface of liposomes [32]. One important limitation with maleimide thiol chemistry is that during storage the thiol group of aptamers may be affected by oxidation and leading to formation of disulfide bond (S-S) between two thiol modified aptamers. These dimeric aptamers are not able to participate in the conjugation reaction with the maleimide functional group of DSPE-PEG_2000_ [30]. Therefore, the use of EDC/NHS reactions increase the product yields and improve in post-insertion method.

Aptamer has some advantages over the antibodies including ease of synthesis and scale-up, low systemic toxicity and lack of immunogenicity [33]. Here, after aptamer conjugation to the lipid, the post-insertion method was recruited to make anti-EpCAM aptamer decorated Caelyx® (ED-lip). Generally, post-insertion technique is simple and effective method for attachment of aptamers to the surface of liposomes and provided a higher rate of aptamer incorporation into liposomes [34].

**Fig. 2:**
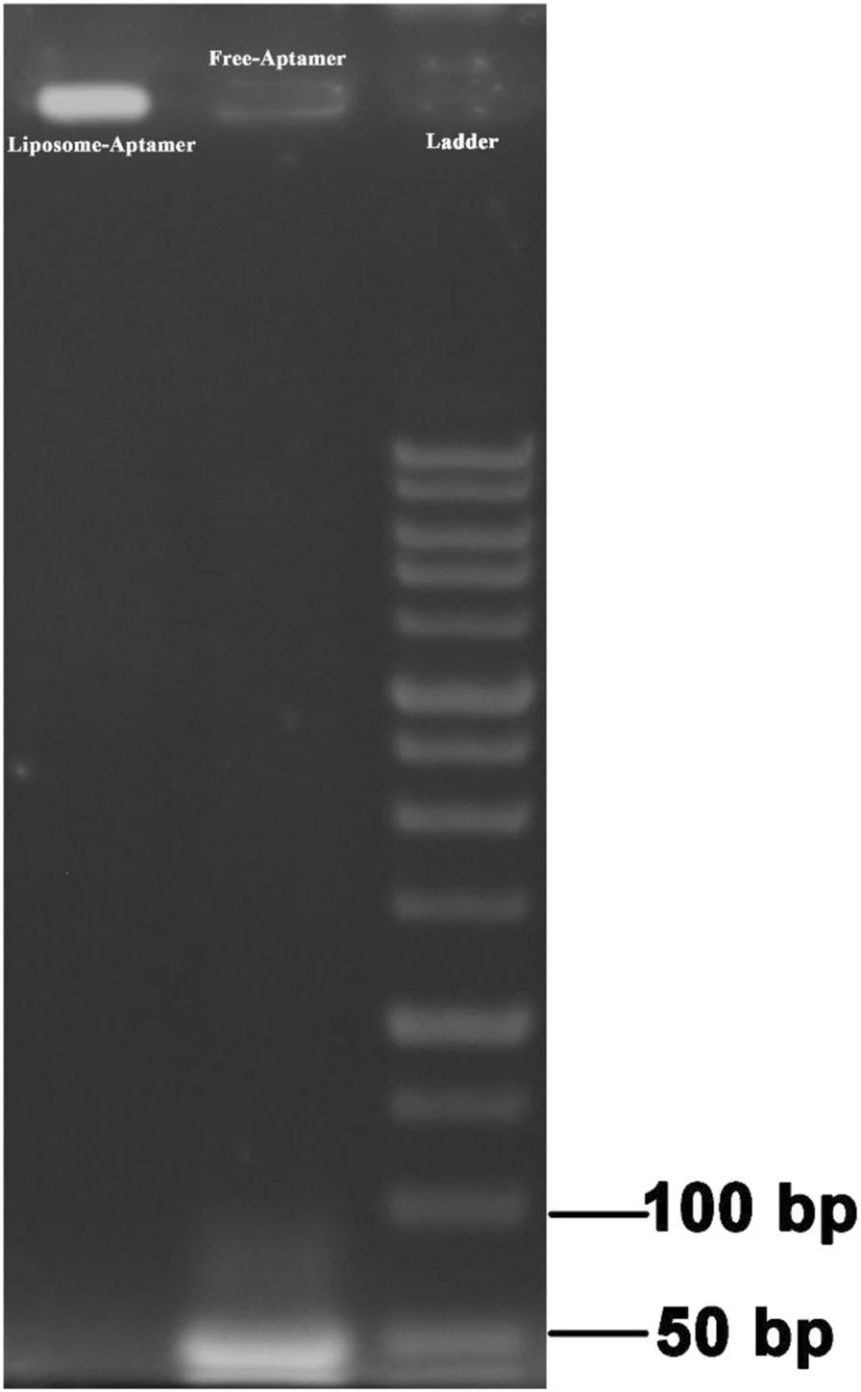
Agarose gel-electrophoresis of ED-lip formulation. Samples are loaded onto the agarose gel. UV light visualized the gel. The wells corresponding to ladder, free aptamer and PL conjugated aptamers are indicated. Lack of corresponding band in liposome-Aptamer indicated the post-insertion confirmation

We used gel electrophoresis mobility shift assay for evaluation of post-insertion of anti-EpCAM aptamer on liposome. As shown in Fig. 2, the negatively charged aptamers migrated in the gel and their band were observed while there is no any counterpart band for ED-lip formulation, because ED-lip trapped in the well line and could not move thorough the gel. These results indicated that aptamers-conjugated micelles were successfully post-inserted into the surface of liposomes.

### Physicochemical Characterization of ED-lip

The physicochemical characterization of Caelyx® and ED-lip was demonstrated in Table 1. The size and charge of prepared formulations revealed that modification of Caelyx® with anti-EpCAM aptamer had no significant effect on the particle size (*p* > 0.05). Liposomal size before insertion of aptamer (Caelyx®) was around 96 nm with PDI of 0.11, after the post-insertion (ED-lip) the size of liposomes partly increased to 117 nm with PDI of 0.14 that have a desirable size for delivery to tumor. The results of previous studies also indicated that incorporation of targeting ligands lead to increase in size and PDI of liposomes [35, 36]. Moreover, the zeta potential of the ED-lip (− 19.25) became more negative than Caelyx® (− 12). It was shown that the RNA-aptamer conjugation into liposome resulted in the decrease in zeta potential of the liposome [37]. The increase in the size and negative zeta potential of the ED-lip could be evidence of successful post-insertion of conjugated aptamers on the surface of liposome [38]. These results are consistent with our previous study that indicated the attachment of aptamer to the surface of Caelyx® leading to slight increase in particle size and the more negative zeta potential in the aptamer functionalized Caelyx® [38, 39]. However, the efficacy of post-insertion should be tested in terms of incubation time and temperature to reach more efficient post-inserted liposome with better size and PDI. The encapsulation efficiency of the Caelyx® and ED-lip were 100% (see Table 1).

**Table 1:** Physicochemical characteristics of ED-lip and Caelyx®. Each value represents as mean ± standard deviation (S.D) (*n* = 3)

Formulations	Size (intensity)	Size (volume)	Size (number)	Z-average^a^ (nm)	PDI^b^	Zeta-potential (mV)	Encapsulation efficiency (%)
Caelyx®	102 ± 2	86 ± 2	76 ± 3	96 ± 2	0.11	-12 ± 0.5	100
ED-lip	110 ± 5 *	90 ± 2	84 ± 2 *	117 ± 2 *	0.14	-19 ± 0.1	100

^a^The size of liposomes (*Z* average)

^b^Polydispersity index

* P < 0.05

The number of aptamer post-inserted to surface of liposome was determined as described [6]. The total amount of phospholipids content of liposomal formulation determined by phosphate assay was 14 mM. Since, the average number lipid molecules in liposome with average size 100 nm is 8× 10^4^ the number of liposomes in each milliliter are nearly 10^14^ [38]. The molecular weight of aptamer was g/mol. The number of DSPE-mPEG_2000_-aptamer was determined based on phosphate assay methods in which moles of phosphate molecules are corresponded to moles of conjugated molecules. Based on these data, the number of aptamer molecules per each ml aliquots solution are 10^15^.

### DOX Release Profile

**Fig. 3:**
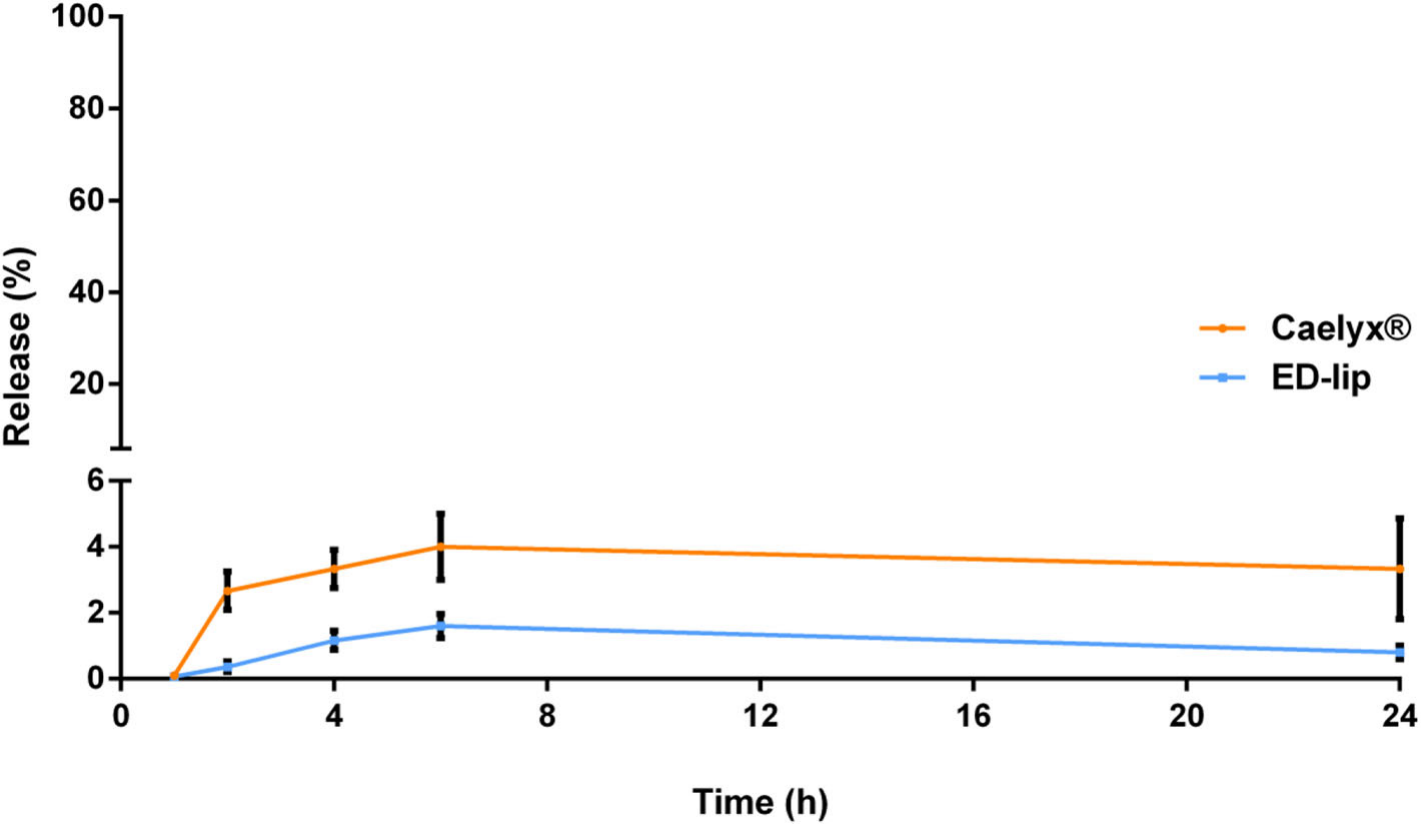
Release study. DOX content leakage profile from Caelyx® and ED-lip at 37 °C at the presence of 50% FBS in dextrose during 24 h of study. Data represented as mean ± standard deviation (SEM) (*n* = 3)

The insertion of aptamer conjugated micelles to the outer surface of Caelyx® may affected release profile of the DOX. Therefore, we evaluated the release of DOX form ED-lip compared to the Caelyx® in 5% dextrose with 50% FBS. This medium could mimic the release behavior of the formulations in the plasma [40]. Figure 3 showed that there is no significant difference in DOX release from Caelyx® and ED-lip formulations during 24 h of study and only the negligible amounts of DOX was released. This is consistent with our previous studies that indicated the insertion of aptamer to the surface of liposome was not affect the membrane stability and release profile of DOX [38, 39]. This is mainly due to the stability of Caelyx® formulation that was formulated using a pH gradient-driven remote loading method [41].

### Cell interaction and Cell Uptake by Fluorescent Microscopy

**Fig. 4:**
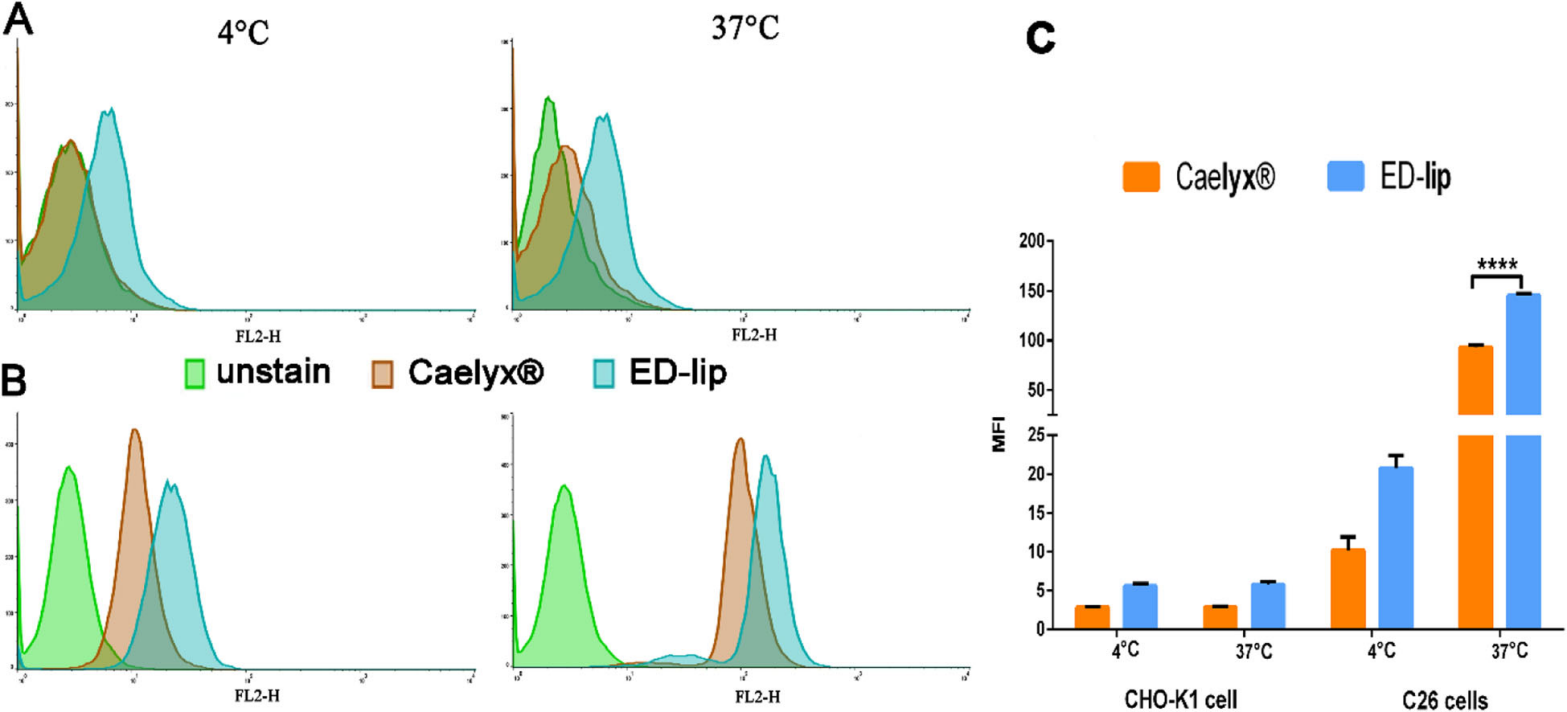
Cell interaction and cell uptake of liposomal formulations evaluated in 4 °C and 37 °C. **a** The CHO-K1 cell interaction of the formulations at 4 °C and 37 °C. **b** The interaction of formulations with C26 cells at 4 °C and 37 °C. **c** The graph demonstrated mean MFI of formulations on CHO-K1 and C26 cells. Data represented as mean ± standard deviation (SEM) (*n* = 3). ****p < 0.0001

**Fig. 5:**
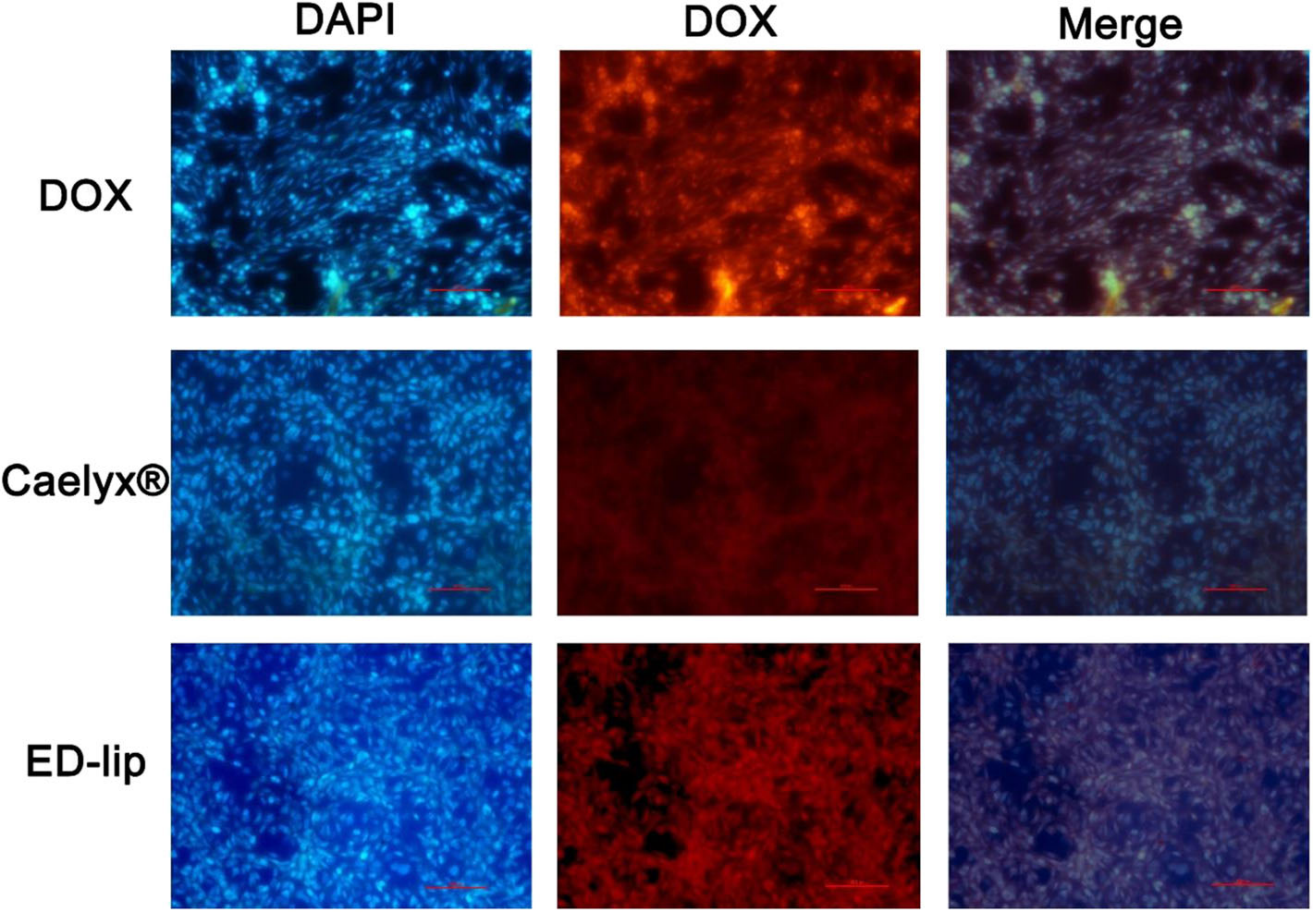
Fluorescent microscopy. The results of cell internalization of DOX on C26 cell lines visualized by fluorescent microscopy. Cells stained with DAPI. Both free DOX and ED-lip have higher level of DOX internalization compared to the Caelyx®. Cells inspected under × 200 magnification

The cell interaction and cell uptake of liposomal formulations were evaluated in 4 °C and 37 °C and has shown in Fig. 4. The evaluation of targeting efficacy of ED-lip indicated that there were no differences among the mean fluorescent intensities (MFIs) of CHO-K1 cells treated with Caelyx® and ED-lip at 4 °C and 37 °C (Fig. 4a, c). However, the data demonstrated that targeted ED-lip considerably had higher uptake by C26 cells compared to Caelyx® at 4 ° and 37 °C (Fig. 4b, d) which was statistically significant at 37 °C (*p* < 0.0001). The ED-lip had the significant uptake in comparison with the Caelyx® (*p* < 0.001). These results indicated that ED-lip enhanced target specificity due to anti-EpCAM aptamer has a more affinity to C26 cell line in comparison to CHO-K1 cells. Free DOX is freely passed through lipid bilayer and enter cell so has the highest cell uptake among the formulations and hence has the highest cytotoxicity. In case of Caelyx®, PEGylation limits the rate of endocytosis and resulted in the decreased cytotoxicity. However, the presence of anti-EpCAM aptamer on the surface of liposome (ED-lip) enhances the rate of internalization of the formulation into the cells and increase its cytotoxicity compare to Caelyx® [38]. The data of fluorescent microscopy demonstrated that difference between cellular uptake of ED-lip and free DOX in C26 cell line at 37 °C was not significant (Fig. 5). However, scaling based on the intensity of internalized DOX depicted in the Table 2 in which both ED-lip and DOX have shown statistically significant differences with Caelyx® in C26 cellular uptake (*p* < 0.001). Although C26 cells express low level of EpCAM on their surface [42], the data of this study suggested that the presence of anti-EpCAM aptamer could enhance the rate of internalization process of liposomes [43].

**Table 2:** Scaling of formulation cell uptake based on doxorubicin fluorescent color

Group no.	Group name	Scaling
1	DOX	4.4 ± 0.325***
2	Caelyx®	1.8 ± 0.162
3	ED-lip	3.72 ± 0.121***

Values are means ± SEM

***p < 0.001 compared with Caelyx® group

### Cytotoxicity Study

**Fig. 6:**
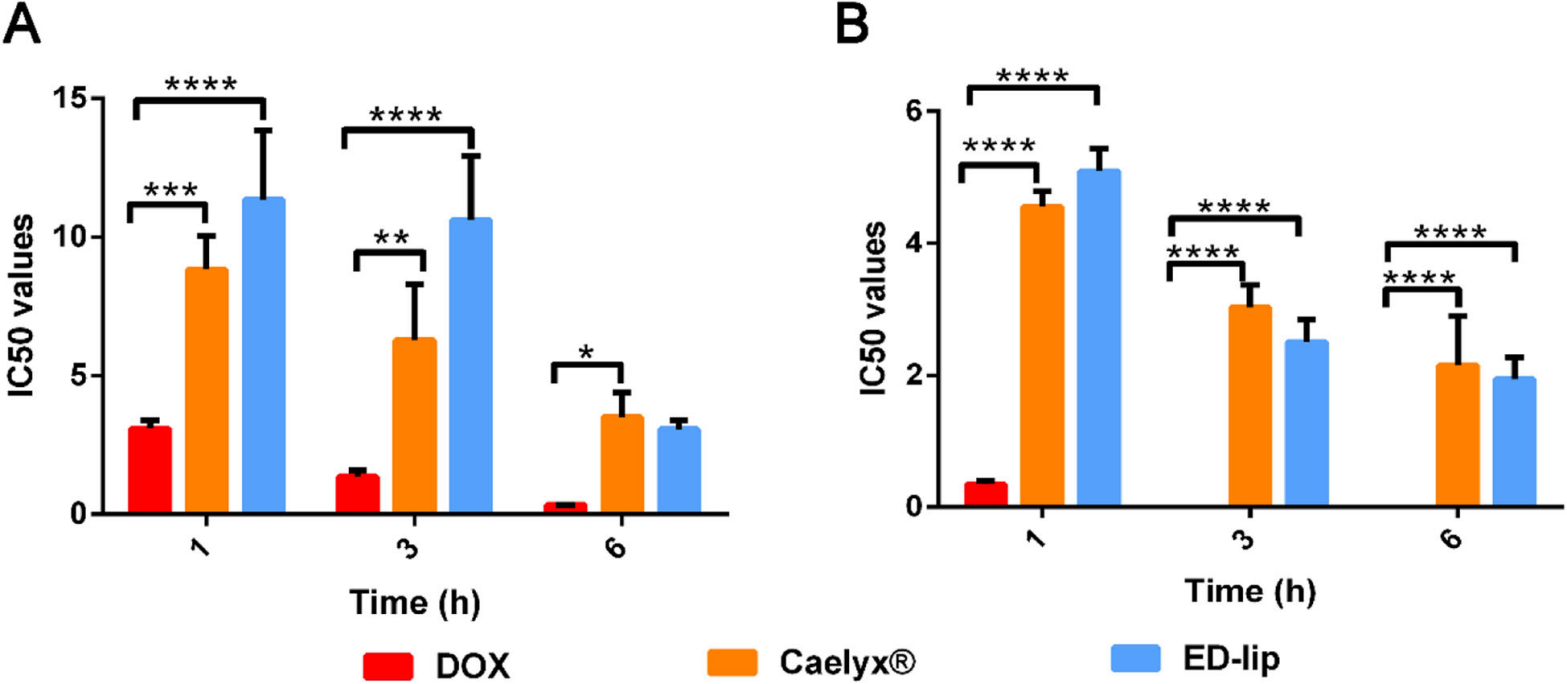
In vitro cytotoxicity effect (IC50) of ED-lip, Caelyx®, and free doxorubicin against CHO cells and C26 cells after different exposure times. Data represented as μg/ml ± standard deviation (SEM) (*n* = 3). *p < 0.05, **p < 0.01, ***p < 0.001, ****p < 0.0001

The cytotoxicity effects of free DOX, Caelyx®, and ED-lip formulations are indicated in Fig. 6. Different concentrations of formulations used to treat cells for 1, 3, and 6 h and allowed to incubate for next 72 h. Data demonstrated that all formulations have effects on cells in time and dose dependent manner. The viability of C26 cells treated with ED-lip formulation decreased compared with Caelyx® treated cells. Since CHO-K1cells, as EpCAM negative cells, show lower response to ED-lip compare to C26 cells, it seem that anti-EpCAM aptamer increased specific delivery of Caelyx® to targeted cells. These results could confirm the specific cellular uptake of ED-lip by C26 cells. These results emphasized the importance use of targeted drug delivery with specific targeting agents to selective delivery of drug to target cells with reducing the side effects of drug by avoiding off-target [44]. As previously reported, the active targeting of Caelyx® with specific targeting ligands such as aptamer and antibody leading to increase active tumor targeting and specific drug delivery to target cells which in turn enhanced the therapeutic efficacy of DOX [35, 39].

### Biodistribution and Pharmacokinetic

**Fig. 7:**
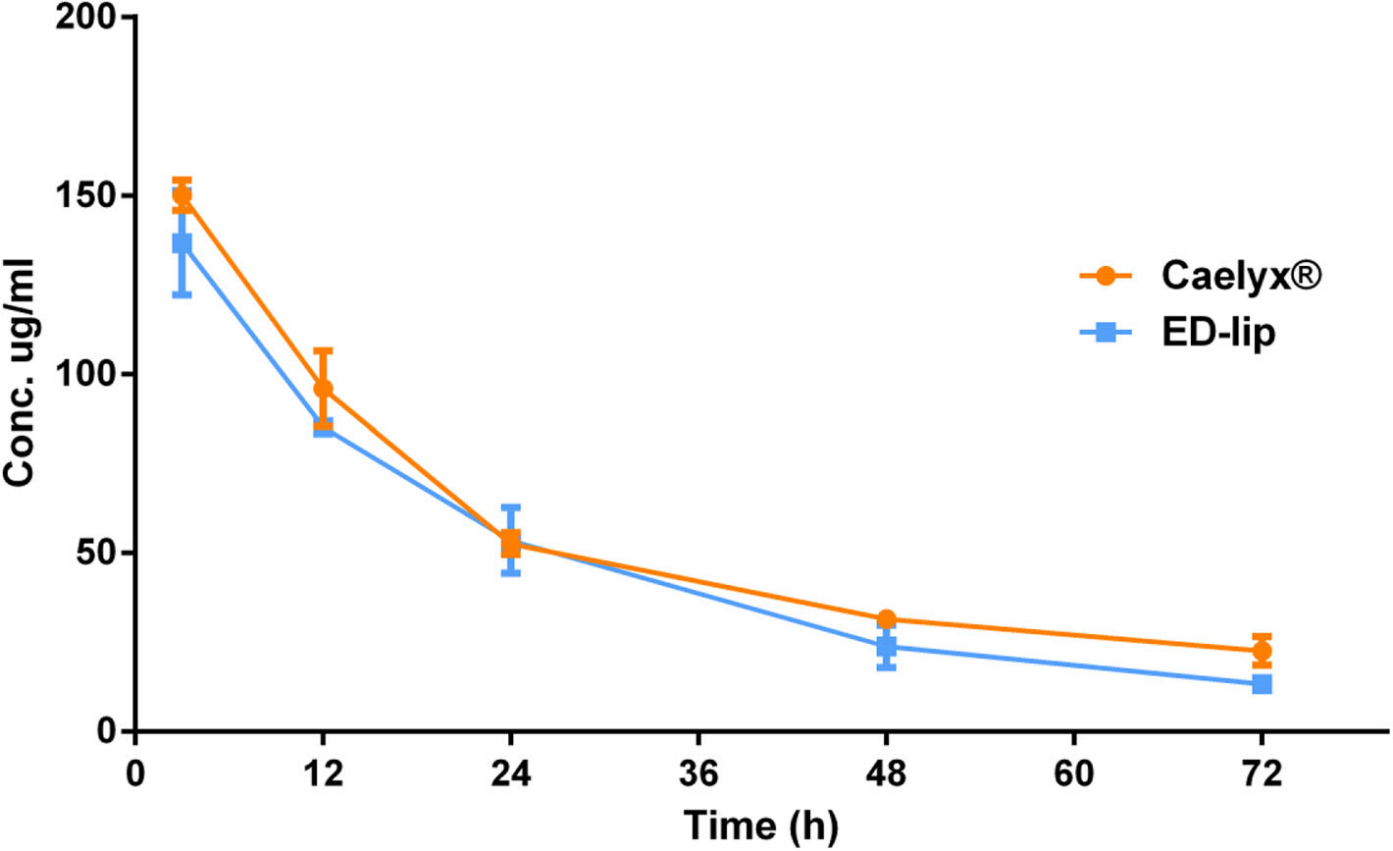
Plasma level of DOX. The results of concentration against time of amount of DOX in blood at 3, 12, 24, 48, and 72 h post-injection. Data represented as mean ± standard deviation (SEM) (*n* = 3)

In order to evaluate how anti-EpCAM aptamer affect the biodistribution of DOX, we injected dose of 10 mg/kg of ED-lip and Caelyx® in mice bearing subcutaneous C26 colon cancer tumors. The DOX concentration in plasma at 3, 12, 24, 48, and 72 h after injection with Caelyx® and ED-lip is shown in Fig. 7. The results show that the behavior of plasma DOX concentrations in both groups was similar and there was no any significant difference between both formulations. As shown in Table 3, the conjugation of anti-EpCAM aptamer to the liposomal surface slightly decreased circulation half time from 39.3 h to 34.2 h and MRT from 47.6 to 42.9 h (see Table 3). The pharmacokinetic parameters indicated that the conjugation of anti-EpCAM aptamer on the liposome slightly decreased t_½_ and MRT which were consistent with previous reports demonstrated that conjugation of aptamer on liposomal surface accelerate clearance of liposomes [38]. Protein adsorption and consequent removal by mononuclear phagocytic system (MPS) could be reason of the acceleration in blood clearance of ligand-conjugated nanoparticles [45].

**Table 3:** Non-compartmental model of pharmacokinetic parameters of Caelyx® and ED-lip administrated i.v. in mice at the single dose of 10 mg/kg

	*t*_½_ (h)	T_max_ (h)	C_max_ (μg/ml)	AUC_0-t_ (μg/ml*h)	AUMC (μg/ml*h^2^)	MRT (h)	Cl (mg/kg)/(μg/ml)/h)	V_d_ (mg/kg)/(μg/ml)
Caelyx®	39.3	3	150.1	4142	258110	47.6	0.0018	0.087
ED-lip	34.2	3	136.7	3926	211668	42.9	0.002	0.087

**Fig. 8.**
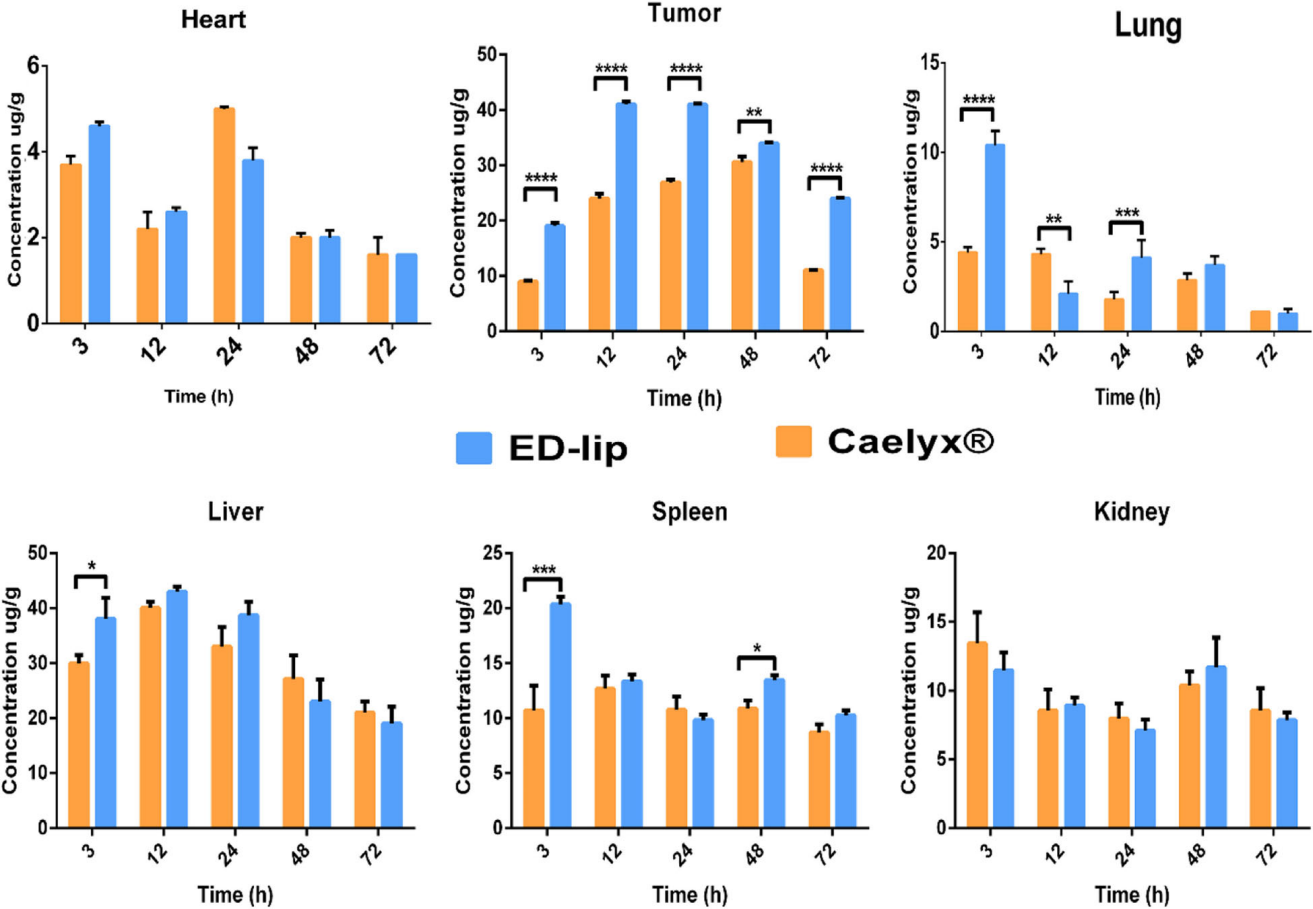
Tissue biodistribution. The results of DOX biodistribution in heart, tumor, liver, lung spleen, and kidney. The concentrations (μg/g) reported at 3, 12, 24, 48, and 72 h post-injection for each organ. Data represented as mean ± standard deviation (SEM) (*n* = 3). *p < 0.05, **p < 0.01, ***p < 0.001, ****p < 0.0001

As shown in Fig. 8, the concentrations of DOX in major harvested organs in groups receiving Caelyx® and ED-lip were compared. The most important side effects of free DOX is the cardiotoxicity which Caelyx® significantly decrease the risk of this adverse effect [46]. The biodistribution of ED-lip in the liver, lung and spleen is significantly higher than Caelyx® at time 3 h. The presence of leaky blood vessels and increase in the size and PDI of the ED-lip after post-insertion may be the reasons for more accumulation of ED-lip in these tissues in earlier times. It was shown that the increase in the size of nanoparticles to 150 nm enhance the liver, lung and spleen accumulation of nanoparticles [47]. Meanwhile, the results of biodistribution study clearly show the EPR mechanism in the accumulation of nanoparticles in the tumor. Figure 8 clearly shows that both ED-lip and Caelyx® gradually accumulate in the tumor site and reaches to a maximum at around 12 h, stay plateau up to 24 h and then decreases at 48 and 72 h, gradually. It is interesting in all the time point of 3, 12, 24, 48, and 72 h; the accumulation of ED-lip in tumor is significantly more than Caelyx®, which could be due to the efficacy of the active targeting with anti-EpCAM aptamer. These results indicated that the attachment of aptamer on liposome surface dose not affected the DOX distribution in kidney. Therefore, it seems anti-EpCAM aptamers effectively promote tumor specific penetration of liposomes which also could be due the overexpression of EpCAM molecules in tumor vascular endothelial cells [48]. Previously, it was indicated that anti-EpCAM aptamers could enhanced tumor penetration in xenograft tumors [49]. The CSCs or TICs are also target of anti-EpCAM therapy. The administration of anti-EpCAM aptamers as targeting ligand in order to target EpCAM showed promising effects in targeting CSCs [22, 50]. Here, it could be suggested that part of the efficient antitumor effect of ED-lip could be due to successful targeting of CSCs.

### In Vivo Anti-Tumor Activity

**Fig. 9:**
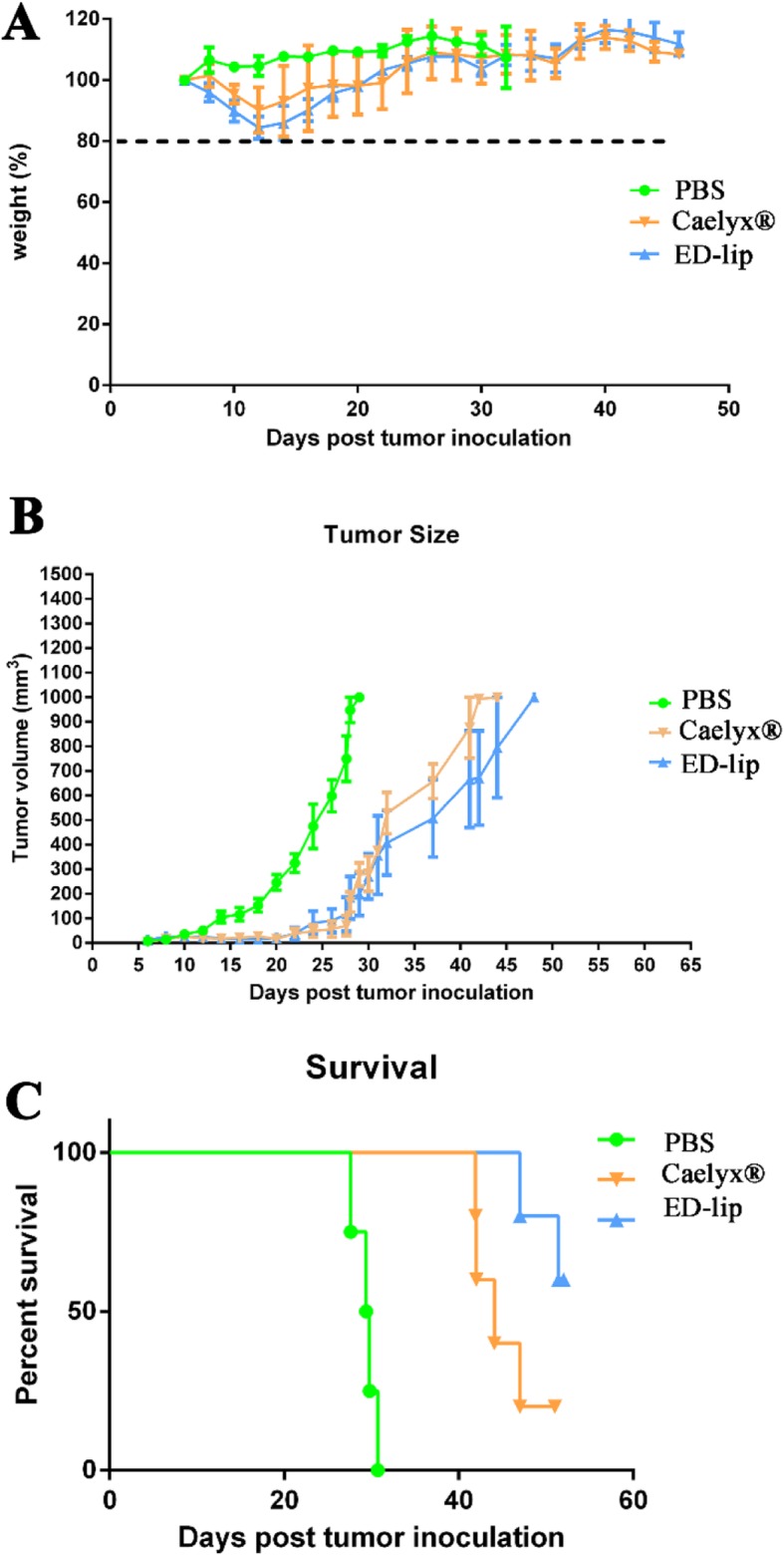
In vivo therapeutic efficacy of formulations in female BALB/c mice bearing C26 colon carcinoma. Mice received IV injection of single dose of formulations (10 mg/kg). **a** Represents the respective weight percentage profile of the BALB/c in each experimental group. **b** Depicts the tumor size follow-ups in BALB/c mice. **c** Shows the survival graph for BALB/c. Data represented as mean ± SD (*n* = 5)

Therapeutic efficacy of ED-lip was evaluated in C26 colon carcinoma tumor model. Tumor size, body weight, and survival were monitored during almost 2 months and results are summarized in Fig. 9 and Table 4. Data indicate that ED-lip has no obvious influence on mice body weight as well as Caelyx® (see Fig. 9a). As shown in Fig. 9b, after intravenous injection of Caelyx® and ED-lip, the tumor growth rate is efficiently inhibited up to day 30 post injection, and there is no significant difference in liposomal groups. After 30 days post-injection, the rate of tumor growth accelerated, however the growth rate in drugs receiving groups was still slower than PBS receiving group. The difference between Caelyx® group and ED-lip was not significant during 30 days of post-injection. The survival results are represented in a Kaplan–Meier plot. Figure 9c shows ED-lip improves survival curve compared with PBS or Caelyx®. The main indicators of survival study were summarized in Table 4. The tumors in three mice of ED-lip group were completely healed so the MST for this group is undefined. Treatment with ED-lip increased TTE from 41.1 to 49.7 days and resulted in effective anti-tumor activity with 90.27% TGD with undefined MST due to complete removal of tumor in three mice (see Table 4).

**Table 4:** Therapeutic efficacy data of Caelyx® and ED-lip in mice bearing C26 tumor

Groups	TTE^a^ (day ± SD)	TGD^b^ (%)	MST^c^ (Day)
PBS	26.146 ± 6.2	0	29.6
Caelyx®	41.102 ± 8.6	68.68	44.1
ED-lip	49.749 ± 2.2	90.27	Undefined^d^

^a^ Time to reach end-point

^b^ Tumor growth delay (in comparison with buffer group)

^c^ Median survival time

^d^ Due to complete curing of 3 mice in ED-lip group it is not possible to calculate MST for this group and reported as undefined

Tumor size data demonstrated that ED-lip could dramatically inhibit tumor growth. The survival analysis results showed that treatment with ED-lip increased MST and TTE. The group receiving ED-lip had a greater TGD% and were more effective compared with Caelyx®. Our findings are consistent with the high level of DOX concentration in the tumor tissue of ED-lip-treated group. Therefore, aptamer-conjugated liposomal DOX improves their penetration and consequently enhanced the drug accumulation in tumor site, which in turn leads to increase in efficacy of Caelyx® and higher TGD% in survival data. Taken together, these finding indicate that anti-EpCAM aptamers could serve as important targeting agent for drug delivery.

## Conclusion

Here, we have surface-functionalized Caelyx® with anti-EpCAM (SYLC3) aptamer via post-insertion (ED-lip). The flow cytometry and fluorescent microscopy showed high level of DOX uptake in C26 cells which indicated that aptamer could enhance the rate of internalization process of ED-lip. The pharmacokinetic data indicated that the post-insertion of DSPE-mPEG-EpCAM did not change the pharmacokinetic of DOX compared to the Caelyx®. However, the tissue biodistribution showed that the more tumor accumulation of ED-lip in comparison with Caelyx® even after 72 h post-injection. We demonstrated that ED-lip had improved therapeutic effects in mice bearing C26 tumors. The improved survival parameters in mice treated with ED-lip, suggest that the EpCAM-targeted-DOX liposome is a promising drug-delivery carrier for the treatment of cancers and merits further investigation.

## Materials and Methods

### Materials

The 5′-Amine-anti-EpCAM DNA aptamer (sequence of 5′ -CACTACAGAGGTTGCGTCTGTCCCACGTTGTCATGGGGGGTTGGCCTG-3′) (SYL3C) was purchased from BIONEER (biotechnology company, Daejeon, South Korea). DSPE-mPEG_2000_-COOH was purchased from Avanti Polar Lipids (Alabaster, AL). Dowex®, 1-Ethyl-3-(3-dimethylaminopropyl) carbodiimide (EDC), N-Hydroxysuccinimide (NHS), penicillin streptomycin and Fluoroshield™ with DAPI were purchased from Sigma-Aldrich (St. Louis, MO). Commercially available caelyx® was purchased from Behestan Darou Company (Tehran, Iran).

### Conjugation of DSPE-mPEG_2000_ to Aptamer

Anti-EpCAM aptamer was linked to DSPE-mPEG_2000_ through covalent binding of the primary amines (-NH2) of anti-EpCAM aptamer to the carboxyl group (–COOH) of DSPE-mPEG_2000_ (Fig. 1). Conjugation was performed via EDC/NHS coupling chemistry [51]. Briefly, DSPE-mPEG_2000_ was dispersed in 2-(N-morpholino)ethanesulfonic acid (MES) buffer (pH 6.5) and EDC/NHS 400 mM EDC and 100 mM NHS were added to the dispersion. The dispersion allowed stirring for 15 min in order to activate carboxyl groups of lipid. Then, anti-EpCAM aptamer was added to the dispersion and stirred for next 2 h in room temperature in dark. The molar ratio of lipid:anti-EpCAM aptamer was 1:1 and the molar ratio of EDC/NHS was 10-fold of lipid.

### Modification of Caelyx® with DSPE-mPEG-Anti-EpCAM Aptamer

ED-lip was synthesized by the post-insertion method. In order to perform post-insertion, DSPE-mPEG-anti-EpCAM aptamer micelles was added to 1 ml of caelyx® for 30 min at 60 °C. The amounts of DSPE-mPEG-EpCAM aptamer were determined according to Bartlett phosphate assay [52]. Based on approximate number of liposome per milliliter of caelyx® which is about 10^14^, the volume of DSPE-mPEG-anti-EpCAM was adjusted to reach 10 aptamer per each liposome [36]. Agarose gel electrophoresis used to confirm post-insertion [39].

### Physicochemical Characterization

Particle size, polydispersity index (PDI), and surface charge were determined by Dynamic Light Scattering instrument (DLS) (Nano-ZS; Malvern, UK). In order to remove free DOX, liposomes were mixed with Dowex® resin and rotated for 60 min and run through Poly-Prep columns (Bio-Rad Laboratories Inc.) for removing the Dowex® [53]. The amounts of DOX in liposomal formulations were determined using LS-45 fluorescence spectrophotometer (Perkin-Elmer, UK), (excitation: emission 485:590 nm).

### Release Study

In order to evaluate the release of DOX, 1 ml of formulation added to the 9 ml dextrose (with 50% fetal bovine serum (FBS)) and at the certain time intervals (0, 1, 2, 4, 6, 12, and 24 h), samples were taken. After removing free DOX with Dowex® resin the amounts of drug remained in the liposomes were determined by fluorescence spectrophotometer and the percentage of release was calculated [39].

### Cell Culture

C26 colon carcinoma and Chinese hamster ovary (CHO-K1) cell lines were purchased from Pasteur Institute of Iran. Cell lines were cultured in RPMI1640 medium supplemented with 10% of FBS obtained from Gibco (Thermos Fisher Scientific, USA) and 100 IU/ml penicillin, and 100 mg/ml streptomycin. The cells were incubated at 37 ^°^C with a 5% CO_2_ and 95% air humidified atmosphere.

### Cell Interaction and Cellular Uptake Assay

Cell interaction and cell uptake of formulations were evaluated in 4 °C and 37 °C, respectively. Two cell lines, CHO-K1 and C26, were selected in this test. The cells seeded in each well of 12-well plates (2.5 × 10^5^ per well). After overnight incubation in 37 °C, treatments added to the cells and plates were placed at 4 °C and 37 °C and incubate for another 3 h. Then cells washed with PBS, and trypsinized. The fluorescence intensity for DOX was determined using flow cytometry (BD FACSCalibur cytometer). The data were analyzed with FlowJo version 7.0 software.

### Fluorescent Microscopy Evaluation

The number of 1 × 10^6^ cells per well C26 Cells were seeded into 6-well plates in which sterile microscopic cover glass were already inserted. After overnight incubation in 37 °C and 5% humidity, cells were treated with free DOX, Caelyx® and ED-lip for 24 h for complete cell uptake [54]. Then cells washed with PBS and fixed with 4% formaldehyde. Cover glasses stained with Fluoroshield™ with DAPI and were mounted on the glass slides. Treatments were performed in triplicate. From each slide, six zones were selected under × 200 magnification field. Intrinsic fluorescent of DOX was used for evaluation of drug cell uptake. Scaling was performed based on the percentages of cells which shown DOX cell uptake in each microscopic filed:

1: 0–20%, 2: 20–40%, 3: 40–60%, 4: 60–80%, and 5: 80–100%

### Evaluation of Cytotoxicity

The IC50 values of free DOX, caelyx®, and ED-lip were determined by MTT assay. In order to do this, CHO-K1 and C26 cells were seeded at density of 5 × 10^3^ cells per well in 96-well plates at 37 °C. After overnight incubation liposomal formulations and free DOX solution were serially diluted in FBS-free medium and added to cell cultures and incubated 1, 3, and 6 h at 37 °C. Then, cells were washed and allowed to incubate 72 h. The optical densities (ODs) were measured using a spectrometric absorbance of 570 nm against a background of 630 nm on Stat-Fax 2100 microplate reader (Awareness Technology Inc. USA). Then the IC50 values were calculated.

### Animal Study

Female BALB/c mice (4–6 weeks, 18–20 g) were kept in separate cages at 22 ± 2 °C and maintained on standard pellet diet and water ad libitum. Intraperitoneal (i.p) injection of ketamine and xylazine (100 mg/kg ketamine and 10 mg/kg xylazine) used to anesthetize the animals [55]. The number of 3 × 10^5^ C26 cells per mouse in 60 μl PBS injected at the right flank, subcutaneously. Two weeks after inoculation when tumor sizes grew about 5 mm^3^, mice were randomly divided into 3 groups (*n* = 3 for biodistribution and *n* = 5 for antitumor study mice per group). All of the experimental protocols were approved by the Mashhad University of Medical Sciences committee for animal ethics and were performed according to the international rules considering the animal rights.

### Biodistribution and Pharmacokinetic Studies

Fourteen days after tumor inoculation, mice were treated with dose of 10 mg/kg of caelyx® and ED-lip intravenously (i.v.) via the tail vain. Control group received 200 μl PBS solution. At certain time-intervals (3, 12, 24, 48, and 72 h) post-injection mice were euthanized and blood samples and tissue samples (liver, spleen, kidney, lung, heart, and tumor) were collected. Then, the concentration of doxorubicin in each sample measured based on fluorescent intensity of each samples using LS-45 fluorescence spectrophotometer (Perkin-Elmer, UK). Doxorubicin concentration of each sample was measured and non-compartmental analysis of the pharmacokinetic parameters were calculated from blood concentration vs. time profiles. Then the parameters including under the concentration-time curve (AUC) and area under the first moment curve (AUMC), half-life (*t*_1/2_), volume of distribution (*V*_d_), *C*_max_, *T*_max,,_ mean residence time (MRT), and clearance (Cl) were calculated.

### In Vivo Antitumor Activity

In order to evaluate antitumor activity, 10 days after tumor inoculation, mice with palpable tumor size were received single i.v. dose of 10 mg/kg Caelyx® and ED-lip. PBS injected in mice which considered as negative control. The parameters including time to reach the endpoint (TTE), percentage of tumor growth delay (TGD), median survival time (MST), and survival were determined. During the study, mice were observed for health and body weight changes. The tumor volume was also measured using a digital caliper and calculated as follows:

$$ \mathrm{Tumor}\ \mathrm{Volume}=\left(\mathrm{Height}\times \mathrm{Length}\times \mathrm{Width}\right)\times 0.52 $$

Considering ethical aspects, mice were removed in case tumor growth was > 1000 mm^3^, or > 20% weight loss or sign of weakness was observed.

### Statistical Analysis

Data were analyzed using GraphPad Prism 6.0 (GraphPad software, Inc., San Diego, CA, USA). Data were demonstrated as mean ± SEM of at least three independent experiments. The *t* test was used in order to evaluate the results of release study, flow cytometry, and biodistribution of the formulations. ANOVA was employed to evaluate the results of fluorescent microcopy and tumor volumes. The Kaplan–Meier method used to calculate the survival parameters include TTE, MST and TGD%. *P* < 0.05 was considered statistically significant.

## Data Availability

All data supporting the conclusions of this article are included within the article.

## Abbreviations

EpCAM: Epithelial cell adhesion molecule
NDDSs: Nano drug delivery systems
DOX: Doxorubicin
EPR: Enhanced permeability and retention
ECM: Extra cellular matrix
CSC: Cancer stem cell
TIC: Tumor initiating cell
MFI: Mean fluorescent intensities
MPS: Mononuclear phagocytic system
EDC: 1-Ethyl-3-(3-dimethylaminopropyl) carbodiimide
NHS: N-hydroxysuccinimide
PDI: Polydispersity index
DLS: Dynamic light scattering instrument
FBS: Fetal bovine serum
OD: Optical density
AUC: Area under the concentration-time curve
AUMC: Area under the first moment curve
*t*_½_: Half-life
*V*_d_: Volume of distribution
MRT: Mean residence time
Cl: Clearance
TTE: Time to reach the endpoint
TGD: Percentage of tumor growth delay
MST: Median survival time
i.p.: Intraperitoneally
i.v.: Intravenously
